# Supplementary material for: Causal Relationship between Aging and Anorexia Nervosa: A White-Matter-Microstructure-Mediated Mendelian Randomization Analysis
Source: Biomedicines. 2024 Aug 16;12(8):1874. doi: 10.3390/biomedicines12081874 (PMC11351342; doi:10.3390/biomedicines12081874)
Supplement: Supplementary file 1 [file biomedicines-12-01874-s001.zip › Supplementary Figure/Figure S4/Singe SNP plot of MR analysis for TL to ubm-b-1784.pdf]

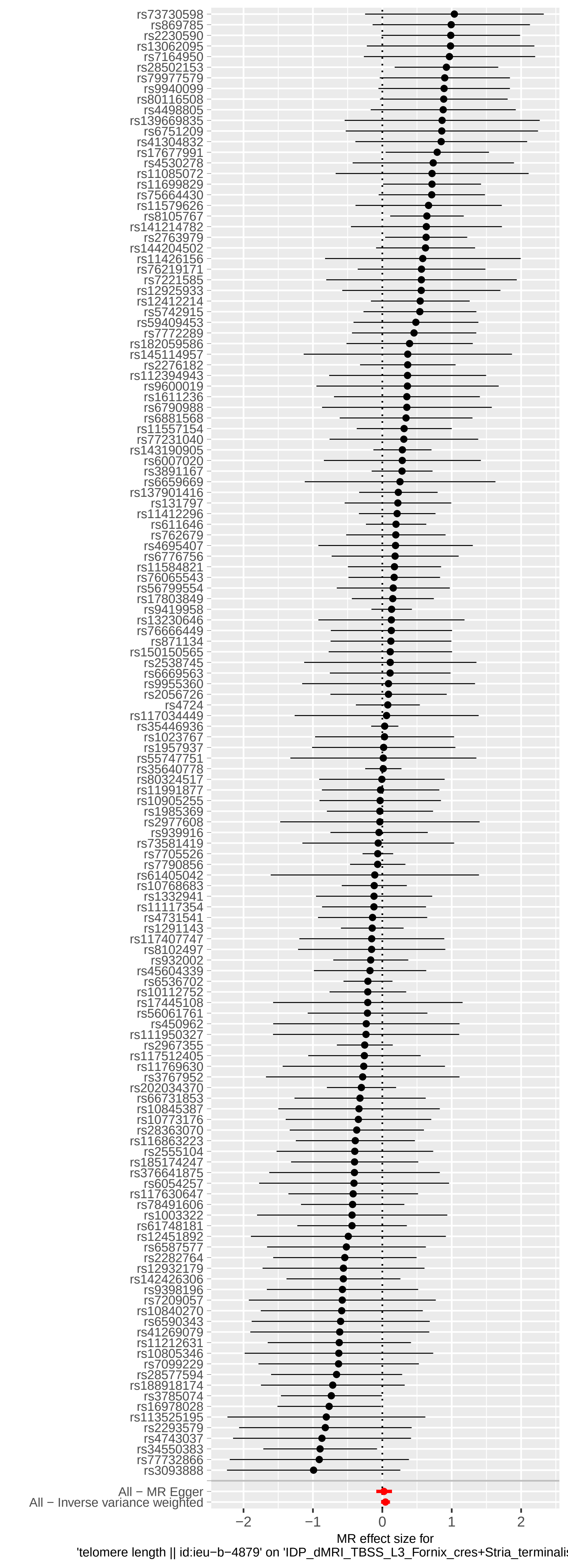
rs73730598
rs869785
rs2230590
rs13062095
rs7164950
rs28502153
rs79977579
rs9940099
rs80116508
rs4498805
rs139669835
rs6751209
rs41304832
rs17677991
rs4530278
rs11085072
rs11699829
rs75664430
rs11579626
rs8105767
rs141214782
rs2763979
rs144204502
rs11426156
rs76219171
rs7221585
rs12925933
rs12412214
rs5742915
rs59409453
rs7772289
rs182059586
rs145114957
rs2276182
rs112394943
rs9600019
rs1611236
rs6790988
rs6881568
rs11557154
rs77231040
rs143190905
rs6007020
rs3891167
rs6659669
rs137901416
rs131797
rs11412296
rs611646
rs762679
rs4695407
rs6776756
rs11584821
rs76065543
rs56799554
rs17803849
rs9419958
rs13230646
rs76666449
rs871134
rs150150565
rs2538745
rs6669563
rs9955360
rs2056726
rs4724
rs117034449
rs35446936
rs1023767
rs1957937
rs55747751
rs35640778
rs80324517
rs11991877
rs10905255
rs1985369
rs2977608
rs939916
rs73581419
rs7705526
rs7790856
rs61405042
rs10768683
rs1332941
rs11117354
rs4731541
rs1291143
rs117407747
rs8102497
rs932002
rs45604339
rs6536702
rs10112752
rs17445108
rs56061761
rs450962
rs111950327
rs2967355
rs117512405
rs11769630
rs3767952
rs202034370
rs66731853
rs10845387
rs10773176
rs28363070
rs116863223
rs2555104
rs185174247
rs376641875
rs6054257
rs117630647
rs78491606
rs1003322
rs61748181
rs12451892
rs6587577
rs2282764
rs12932179
rs142426306
rs9398196
rs7209057
rs10840270
rs6590343
rs41269079
rs11212631
rs10805346
rs7099229
rs28577594
rs188918174
rs3785074
rs16978028
rs113525195
rs2293579
rs4743037
rs34550383
rs77732866
rs3093888
All − MR Egger
All − Inverse variance weighted
−2
−1
0
1
2
MR effect size for
'telomere length || id:ieu−b−4879' on 'IDP_dMRI_TBSS_L3_Fornix_cres+Stria_terminalis
